# Faculty of Comparative Medicine, McGill University, Montreal

**Published:** 1890-09

**Authors:** 


*Faculty of Comparative Medicine, McGill University, Montreal.*—Professor Mills will deliver a special course on canine medicine; also on breeding and management of the kennel, and Professor D. McEachman, a special course on the different breeds, breeding and management of horses and cattle on farms and ranches; also, embracing inspection and transportation of live stock by railroads and steamers. A text-book on "Animal Physiology, Specially Written for Students of Comparative Medicine and Veterinary Science," now in press, will be in circulation by October 1st. The degree D.V.S. *honoris causa* on the dean. The degree D.V.S. in course on Professors Baker and C. McEachnan, and after having fulfilled the requirements and passed the examination on Professor Wesley Mills, M.A., M.D. F. W. Skaife, D.V.S., received, after examination, the M.R.C. V.S. at London.

